# Supplementary material for: Phf8 histone demethylase deficiency causes cognitive impairments through the mTOR pathway
Source: Nat Commun. 2018 Jan 9;9:114. doi: 10.1038/s41467-017-02531-y (PMC5760733; doi:10.1038/s41467-017-02531-y)
Supplement: Supplementary file 1 — Supplementary Information [file 41467_2017_2531_MOESM1_ESM.pdf]

Supplementary figure 1

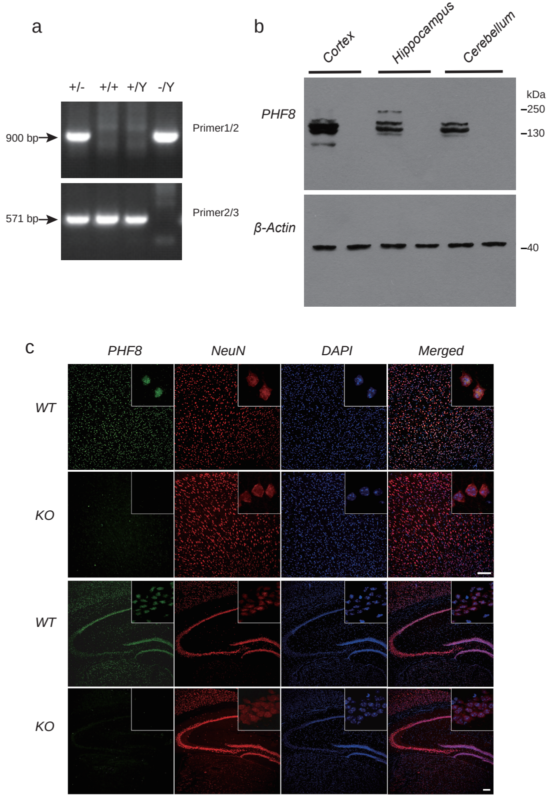

Supplementary figure 1. Targeted disruptions of the catalytic JmjC domain of PHF8 were detected by DNA and Protein analysis.

(a) Three primers were used to analyze the DNA of the mice. For male mice: +/Y, using primer 1/2 and primer 2/3, 571 bp bands were obtained; -/Y, produced a 900 bp band using primer 1/2, but no products were obtained using primer 2/3. For female mice: +/-, 900 bp and 571 bands were obtained using primer 1/2 and primer 2/3, +/+, a 571bp band was obtained with both pair of primers.

(b) Western blotting results show PHF8 protein expression in the cortex, hippocampus and cerebellum in WT and KO mice. β-Actin was used as a control.

(c) PHF8 is expressed in the prefrontal cortex and hippocampus in WT mice, and defect in Phf8 KO mice (green: PHF8, red: NeuN, blue: DAPI). Scale bars, 200μm.

Supplementary figure 2

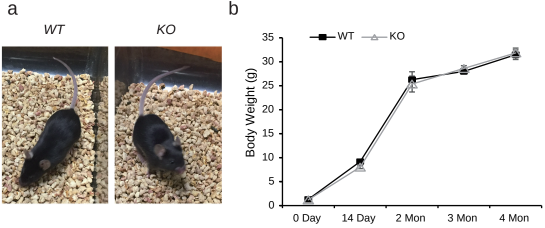

Supplementary figure 2. Non gross morphological differences are found in Phf8 knock out mice.

(a) The WT and Phf8 knockout mice showed no gross morphological differences.

(b) No differences are found in the body weight of Phf8 KO mice and their WT littermates at different age stages.

Supplementary figure 3

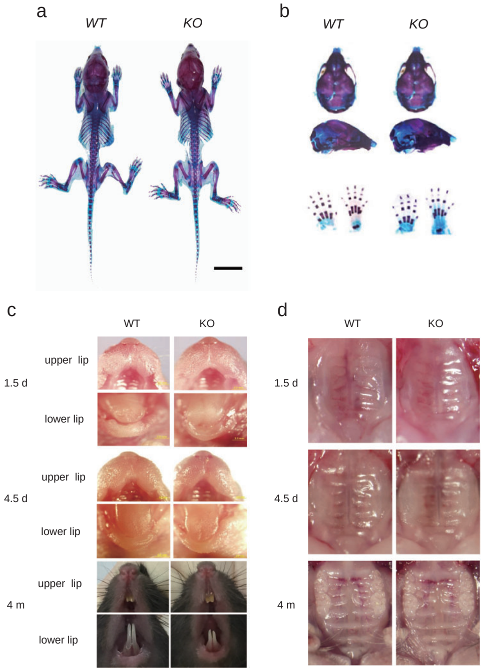

Supplementary figure 3. Both Phf8 KO and WT mice show normal skeleton, lips and palates.

(a) Alizarin red (a marker of bone mineralization) and alcian blue (a marker of cartilage) stained the overall skeleton of the Phf8 KO mice and their WT littermates. Scale bar, 1cm.

(b) Alizarin red and alcian blue stained the skeleton of the skull (up) and extremities (bottom).

(c-d) No differences are found between Phf8 KO and WT mice in their lips (c) or palates (d) at ages 1.5 days, 4.5 days or 4 months.

Supplementay Figure 4

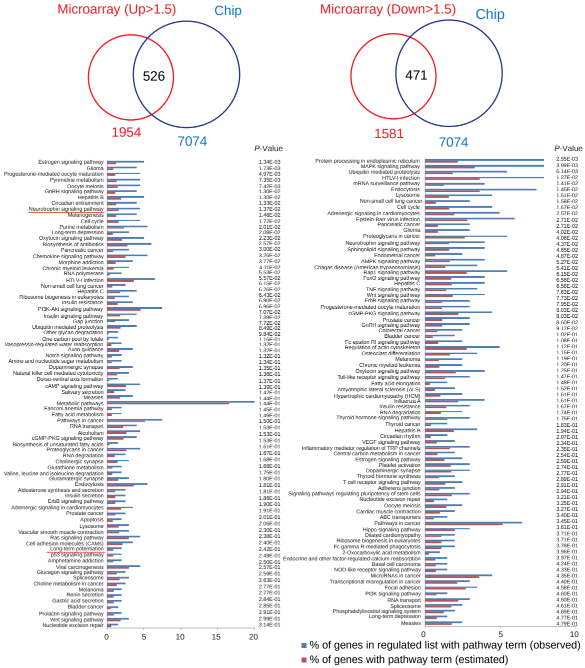

Supplementary figure 4. Venn diagram of overlap between differentially expressed and PHF8-binding genes.

Venn diagram showing the overlap between the differentially expressed genes identified from microarray analysis and PHF8-binding genes screened by CHIP-seq (chromatin immunoprecipitation followed by sequencing) analysis in the literature. The gene ontology (GO) analysis of overlapping data (Up > 1.5, 1.5 fold increase of mRNA expression in Phf8 KO mice as compared to their WT littermate) identifies a set of upregulated and downregulated genes. The red line highlights the pathway of neurotrophin signaling and long-term potentiation (LTP).

Supplementary figure 5

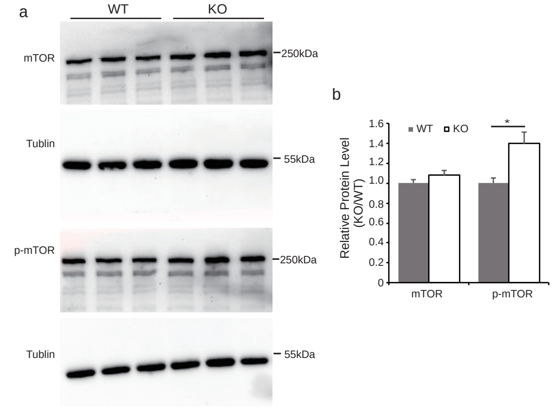

Supplementary figure 5. mTOR and p-mTOR expression in Phf8-dificient hippocampus and their littermates.

(a) Western blotting confirms the hyperactivity of mTOR. The total mTOR has no difference between the KO and WT group, however, phosphorylated mTOR level in PHF8-deficient hippocampus is higher than the wild type mice.

(b) The statistical quantification is shown in the panel (Unpaired t test,*$p<0.05$,n=3).

Supplementary figure 6

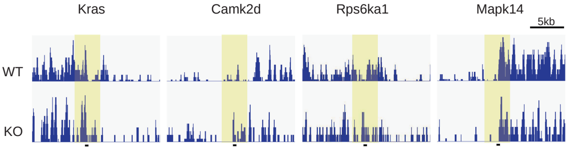

Supplementary figure 6. Chip seqence analysis show increased H4K20me1 binding at the TSS of Kras, Camk2d and Rps6ka1.

Normalized reads show the fold enrichment of H4K20me1 at Kras, Camk2d, Rps6ka1, and MAPK14. H4K20me1 binding at the TSS of Kras, Camk2d and Rps6ka1 were increased after PHF8 Knock out. High lights show region of TSS±2 kb. Black boxes show the TSS.

Supplementary figure 7

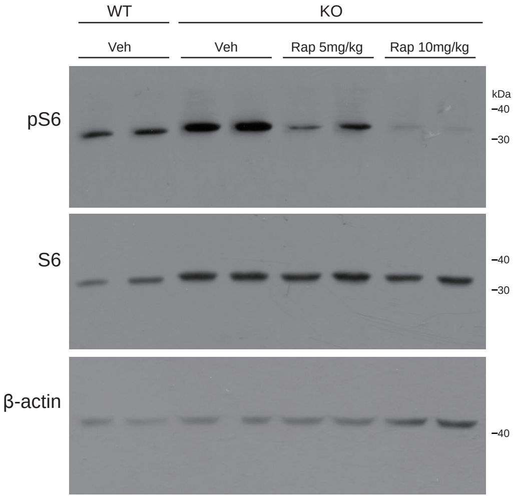

Supplementary figure 7. Rapamycin treatment could inhibit S6 phosphorylation expression in Phf8 KO mice.

The inhibition of S6 phosphorylation was analyzed via western blotting after different doses of rapamycin treatment. Rapamycin at 5 mg/kg was sufficient to inhibit S6 phosphorylation, and was used for all rescue treatment tests.

Supplementary figure 8

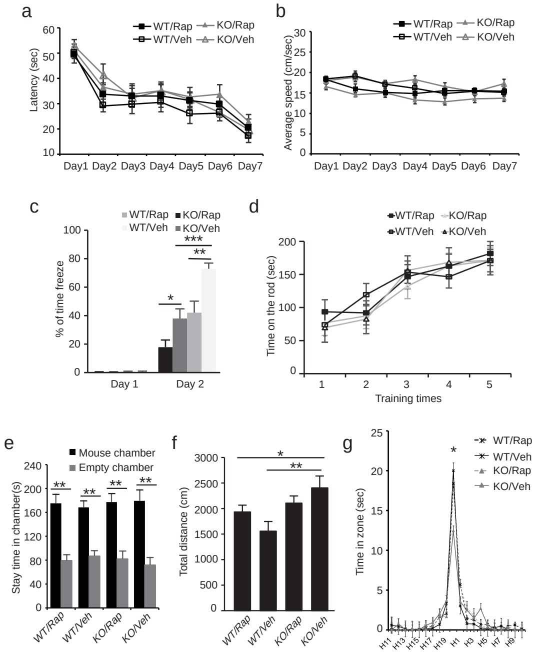

Supplementary figure 8. Other behavior tests after rapamycin treatment.

(a) The escape latency time to reach the hidden platform (WT/Veh, n = 12; WT/Rap, n = 11; KO/Veh, n = 12; KO/Rap, n = 11; WT/Veh vs KO/Veh p value=0.0098,Two way ANOVA).

(b) Average swimming speed during the 7-day training ,no difference are shown among the four groups(WT/Veh, n = 12; WT/Rap, n = 11; KO/Veh, n = 12; KO/Rap, n = 11; unpaired two-tailed t-test).

(c) In contextual fear conditioning test, the percentage of freezing time in the WT/Veh group is much higher than in other three groups (WT/Veh, 72.9 ± 4.01%, n = 10; WT/Rap, 42.1 ± 8.11%, n = 14; KO/Veh, 38.0 ± 6.79%, n = 11; KO/Rap, 17.85 ± 5.05%, n = 13. Unpaired two-tailed t-test, $^{*}p < 0.05$, $^{**}p < 0.01$, $^{***}p < 0.001$).

(d) In the Rota-rod test, the latency to falling down was measured. No differences are shown among the 4 groups.

(e) In the sociability test, the stay time in mouse chamber and empty chamber were recorded (WT/Rap, n = 11; WT/Veh, n = 13; KO/Rap, n = 10; KO/Veh, n = 10; unpaired two-tailed t-test, $^{**}p < 0.01$).

(f) In open field test, the total distance travelled in 20 minutes was measured in each group. Rapamycin treatment has no obvious therapeutic effects on the Phf8 KO mice. (WT/Veh, 1557.53 ± 188.41 cm, n = 12; WT/Rap, 1933.16 ± 133.24 cm, n = 14; KO/Veh, 2405.20 ± 232.31 cm, n = 11; KO/Rap, 2106.35 ± 140.64 cm, n = 13. Unpaired two-tailed t-test, $^{*}p < 0.05$, $^{**}p < 0.01$).

(g) Time travel in each zone around holes during the probe trial (90s) (WT/Rap, n = 11; WT/Veh, n = 12; KO/Rap, n = 10; KO/Veh, n = 10; unpaired two-tailed t-test, $^{*}p < 0.05$)

Supplementary figure 9

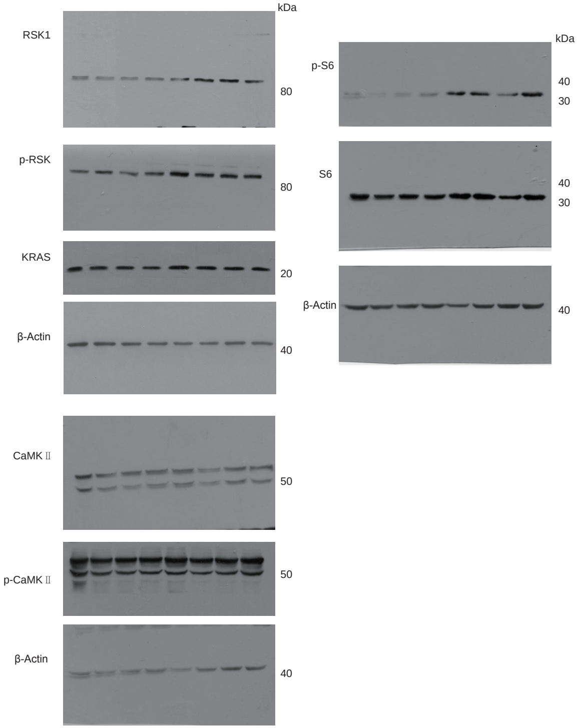

Supplementary Figure 9. Uncropped Western blots of figure 3b and figure 3c.

Supplementary table 1. Neurotrophin signaling pathway related genes

Probe_Set_ID	Fold_change	Regulation	Gene Symbol	Gene Title
1416896_at	1.790323	up	Rps6ka1	ribosomal protein S6 kinase polypeptide 1
1420261_at	3.563447	up	PSEN1	presenilin 1
1422659_at	2.061575	up	Camk2d	calcium/calmodulin-dependent protein kinase II, delta
1426229_s_at	2.1034	up	Kras	v-Ki-ras2 Kirsten rat sarcoma viral oncogene homolog
1418508_a_at	1.865441	up	GRB2	growth factor receptor bound protein 2
1431542_at	2.669818	up	PSEN2	presenilin 2
1450326_at	2.2623	up	SHC3	src homology 2 domain-containing transforming protein C3
1444162_at	4.816175	up	FRS2	fibroblast growth factor receptor substrate 2
1438825_at	17.00923	up	CALM1	calmodulin 1

Supplementary table 2. LTP pathway related genes

Probe_Set_ID	Fold_change	Regulation	Gene Symbol	Gene Title
1416896_at	1.768468	up	Rps6ka1	ribosomal protein S6 kinase polypeptide 1
1422659_at	1.503502	up	Camk2d	calcium/calmodulin-dependent protein kinase II, delta
1426229_s_at	1.72347	up	Kras	v-Ki-ras2 Kirsten rat sarcoma viral oncogene homolog

Supplementary table 3. Primers for quantitative real-time PCR

Primer name	Primer sequence	Primer efficiency
Camk2d-F	GTGGCGAACTGTTTGAAGAC	1.06
Camk2d-R	AATTCTCAGGCTTCAGGTCC	
Rps6ka1-F	GTCTCGGTGTAGCAGCAG	0.97
Rps6ka1-R	GAAGTCTGGCCATTCTCCG	
Kras-F	ATGTGCCTATGGTCCTGGTA	1.00
Kras-R	CATCGTCAACACCCTGTCT	
mus GADPH-F	TGAAGCAGGCATCTGAGGG	0.99
mus GADPH-R	CGAAGGTGGAAGAGTGGGAG	

Supplementary table 4. Primers for ChIP-qPCR

Primer name	Primer sequence	Primer efficiency
Kras 1F	TCCCACCACTAGGAGGTATG	0.90
Kras 1R	CTAGCTTACTCCCGTTGCTC	
Kras 2F	CTGTAAGGGCTGTGACATTG	0.89
Kras 2R	TGTTTTCACGACACAAAGCC	
Kras 3F	GCAAGGTTACTTGGCATGTT	0.99
Kras3R	ACCCCTCAGATTTTCAAGGA	
Cdmk2d 1F	ATCAAGATCCC AGGGGAGAAAA	0.94
Cdmk2d 1R	CCCCACCTCTGTCTTTGCTTT	
Cdmk2d 2F	CTTTGAGGAGCTCGGAAAGT	0.88
Cdmk2d 2R	AAGGTTTCCTTCCCTGTGTG	
Cdmk2d 3F	GAGTCTCATGGGGTGTCTTG	0.91
Cdmk2d 3R	AGCAGTGCTACTGAGCATTT	
Rps6ka1 1F	TCCTCCAATGTTGGGGTTG	0.92
Rps6ka1 1R	ATGGCACTGTGGATAAAGCA	
Rps6ka1 2F	ACTCACAGCTTTACCCTAGC	0.95
Rps6ka1 2R	GGAAATGTGACAGTCTCCCC	
Rps6ka1 3F	CAAAGGTGAGGGAGCTTTCA	0.9 0
Rps6ka1 3R	TCACCAATCATTCCGTAGGC	
MAPK 14 1F	CTCACCCCTGGTATCTTCCT	1.09
MAPK 14 1R	ACTCTGTTTTACCGTGGAGG	
MAPK 14 2F	CTGTAGGGCAAAGCC TGAC	1.03
MAPK 14 2R	GGGGTGCTGCTAATTCAAGT	
MAPK 14 3F	CTCTGGGAGGCTGTTTTAGT	0.96
MAPK 14 3R	GCAAAGGCCTGATTATGAGC	
